# Dispositional and situational empathy in Parkinson's disease and their relationship with cognition

**DOI:** 10.3389/fpsyg.2025.1654067

**Published:** 2025-09-04

**Authors:** Laura Alonso-Recio, Liz Mendoza, África Pérez, Sandra Rubio, Juan Manuel Serrano

**Affiliations:** Department of Biological and Health Psychology, Faculty of Psychology, Autonomous University of Madrid, Madrid, Spain

**Keywords:** cognitive decline, dispositional empathy, empathy, Parkinson's disease, situational empathy, social cognition

## Abstract

**Introduction:**

Parkinson's disease (PD) affects not only motor function but also social cognition, particularly empathy. While most studies focus on dispositional empathy—an automatic, stable trait measured by self-report—situational empathy, assessed in specific contexts, has been barely explored. The relationship between these empathy types and their link to cognitive functioning in PD are largely unknown. This study examines dispositional and situational empathy in PD patients, considering cognitive impairment as a moderating factor.

**Method:**

The sample included 31 cognitively preserved PD patients (MoCA ≥ 26), 39 cognitively impaired PD patients (MoCA < 26), and 33 age-matched healthy controls. Dispositional empathy was assessed with the Interpersonal Reactivity Index. Situational empathy was evaluated through a behavioral task where participants viewed dynamic emotional faces paired with emotionally charged sentences, and selected the emotion they felt. A comprehensive neuropsychological battery assessed cognitive functioning.

**Results:**

No group differences emerged in dispositional empathy. However, cognitively impaired PD patients showed poorer situational empathy compared to the other groups. No significant correlation was found between dispositional and situational empathy, nor consistent correlations between empathy and specific cognitive processes.

**Discussion:**

Findings show that PD affects empathy unevenly: dispositional empathy is preserved, but situational empathy declines with cognitive impairment. This suggests that empathy deficits depend on task complexity and overall cognitive status, not just isolated functions. Since situational empathy requires real-time processing of emotional and contextual cues, it is especially sensitive to cognitive decline. These results highlight the need for comprehensive assessments to reflect PD's neurocognitive variability.

## 1 Introduction

Parkinson's disease (PD) is a progressive neurodegenerative disorder clinically diagnosed by the presence of characteristic motor symptoms such as resting tremor, bradykinesia, and rigidity (Leite Silva et al., 2023). However, increasing evidence highlights that PD is also associated with significant non-motor symptoms, including impairments in social cognition (SC), which affect ~20% of patients (Christidi et al., 2018; Dodich et al., 2021; Romosan et al., 2019). SC is a complex cognitive domain that encompasses a range of psychological processes crucial for human interaction, such as recognizing social and emotional cues, processing others' beliefs and intentions, maintaining social knowledge, and generating appropriate behavioral responses (Multani et al., 2019). Despite its relevance, SC has received relatively limited attention in PD research (Chen et al., 2022). This is particularly concerning, as social dysfunction can precede the full clinical onset of PD by several years, emerging before the appearance of overt motor symptoms (Chen et al., 2022; Yu and Wu, 2013). Furthermore, SC plays a fundamental role in navigating social environments and maintaining interpersonal relationships, with deficits in this domain significantly impacting mental health, wellbeing, and overall quality of life (Christidi et al., 2018; Trompeta et al., 2021).

A key component of SC is empathy, which refers to the ability to understand and share the thoughts and emotions of others while also engaging emotionally and caring about their wellbeing (Han et al., 2025). Previous research suggests that patients with PD may exhibit reduced empathic responses, potentially due to neural damage associated with the disease (Coundouris et al., 2020). Empathy relies on the participation of integration of multiple structures including the prefrontal cortex, the anterior cingulate cortex (ACC), the amygdala, the insula, and the basal ganglia—all of which are affected in PD (Rankin et al., 2006).

Most existing research relies on self-report measures, such as the Interpersonal Reactivity Index (IRI; Davis, 1983) or the Empathy Quotient (EQ; Baron-Cohen and Wheelwright, 2004), in which patients are interrogated using empathy-inducing hypothetical situations (de Lima and Osório, 2021). These self-reported measures provide information on dispositional empathy, which refers to a person's stable character trait that exists outside of particular situations (Aparicio-Flores et al., 2020; Cuff et al., 2014). It represents an individual's response tendency to adopt others' perspectives across various contexts, and is considered relatively consistent over time. Dispositional empathy is defined as the automatic generation of empathetic reactions, and is regarded as an innate character trait that cannot be taught (Rameson et al., 2012). Some authors distinguish two components within dispositional empathy, one affective and one cognitive (Davis, 1983). Affective empathy includes emotions directed toward others and cognitive empathy involves the ability to take the perspective of another person's mental state (Mattan et al., 2016). The results obtained in PD patients using these dispositional empathy measures have been very diverse. While some studies report no empathy deficits in PD (Alonso-Recio et al., 2021; Schwartz and Pell, 2017), others find generalized impairments—especially in advanced disease stages (Dodich et al., 2021; Narme et al., 2013; Pomponi et al., 2016)—or specific deficits in cognitive empathy while affective empathy remains intact (Coundouris et al., 2020; Multani et al., 2019; Schmidt et al., 2017).

Nevertheless, empathy can also be measured through situational tasks that assess empathic reactions in specific or immediate contexts (Auné et al., 2015; Cuff et al., 2014; Fernández-Pinto et al., 2008). Particularly, it can be evaluated in stimulated experimental settings by asking subjects to report their feelings immediately after exposure to a situation designed to elicit empathic responses. Therefore, in contrast to dispositional empathy, situational empathy is influenced by an individual's social ability in a particular context, and it manifests in a manner that is more contingent on the circumstances (Aparicio-Flores et al., 2020). In PD, we identified only one study that specifically focuses on situational empathy. In particular, we have identified a study focusing on the concept of pain empathy, in which patients had a decreased empathic response to situations involving the pain of others (Hu et al., 2021). No studies were found that analyzed the capacity of PD patients to respond empathetically to scenarios eliciting basic emotions such as happiness, sadness, anger or fear.

While the distinction between dispositional and situational empathy is less frequently emphasized in clinical literature compared to the more widely recognized dimensions of affective and cognitive empathy, it does not conflict with them. Rather, dispositional and situational empathy refer to the temporal and contextual framing of empathic responses, and both may encompass cognitive and affective components. Therefore, the two frameworks can be viewed as complementary. Focusing on the distinction between dispositional and situational empathy, it is important to examine how these two forms differ. Understanding this distinction is crucial, as the specific empathic emotion experienced in a given context is not necessarily aligned with an individual's general tendency to empathize (Batson et al., 1987; Fabi et al., 2019). Along these lines, neuroimaging studies suggest that brain activity related to empathy correlates more strongly with situational rather than dispositional empathy scores (Bufalari and Ionta, 2013; Lamm et al., 2011; Saarela et al., 2007; Singer et al., 2004). Research examining the relationship between dispositional and situational empathy in healthy individuals has found only modest correlations (Fabi et al., 2019). A meta-analysis reported a mean effect size of *r* = 0.35 for the association between dispositional empathic concern and situation-specific empathic responses (Zickfeld et al., 2017). These moderate correlations suggest that while related, dispositional and situational empathy represent distinct aspects of empathic functioning. Some researchers argue that empathy is generally more situational than dispositional, noting that self-report measures of dispositional empathy may be susceptible to social desirability bias, self-awareness and verbal expression difficulties (Jauniaux et al., 2020; Zhou et al., 2003). Consequently, combining measures of both situational and dispositional empathy may provide a more comprehensive understanding of empathic capacity. In PD, there is a lack of research exploring the relationship between dispositional and situational empathy in PD, leaving a critical gap in our understanding of how these two dimensions interact in the context of PD.

Another important debate about empathy abilities in PD refers to the dependence on, or independence from cognition, both general and specific processes. Regarding the relationship between empathy and other cognitive domains, the most studied is with executive functions. They have been related to SC abilities in PD (Narme et al., 2013; Yu and Wu, 2013). In fact, lower scores in different SC tasks, such as social perception or theory of mind, have been related to attention, working memory, and other executive function impairments (Assogna et al., 2010; Narme et al., 2011). Nevertheless, other studies have failed to find this link (Alonso-Recio et al., 2014; Bodden et al., 2010; Herrera et al., 2011; Pietschnig et al., 2016; Roca et al., 2010). Moreover, to date, only one study has examined specifically the relationship between empathy and cognition in PD, observing sporadic and non-significant correlations between empathy scores and several cognitive functions (Schmidt et al., 2017). The authors of the study posit that the majority of patients showed only slight cognitive impairments, and that the absence of significant results may be attributable to a ceiling effect in cognitive measures. For their part, Alonso-Recio et al. (2021) studied the relationship between empathy and general cognitive status (measured by the MoCA) of two groups of PD patients (with and without cognitive impairment) and a group of healthy individuals, finding no differences between them. However, changes in empathic ability have been found in people with PD and dementia, with similar patterns to those observed in other neurodegenerative conditions such as Alzheimer's disease (Martinez et al., 2018).

In summary, despite the relevance of empathy in PD, it remains unclear how dispositional and, particularly, situational empathy abilities are affected in this population. Moreover, the relationship between these two types of empathy has not yet been assessed in PD patients. Accordingly, this study aimed to examine both dispositional and situational empathy in individuals with PD. Moreover, given the potential influence of other cognitive functions on these abilities, this research also examined the relationships between both types of empathy measures and various cognitive processes, including processing speed, visuospatial abilities, memory, language, and executive functions. To this end, the present study compared a group of PD patients with cognitive impairment, a group of cognitively-preserved PD patients and an equivalent HC group.

## 2 Materials and methods

### 2.1 Participants

The experimental sample consisted of 70 individuals diagnosed with idiopathic Parkinson's disease by neurologists specialized in movement disorders, following international diagnostic criteria (Hughes et al., 1992). PD patients were recruited from three institutions in Madrid (the Parkinson's Association of Alcorcón and surrounding municipalities, the Parkinson's Association of San Sebastián de los Reyes, and the Parkinson's Association of Madrid) and one in Asturias (the Parkinson's Association of Asturias). Based on their scores on the Montreal Cognitive Assessment (MoCA; Nasreddine et al., 2005), participants were classified as cognitively intact (PD_CogInt; MoCA ≥ 26, *n* = 31) or cognitively impaired (PD_CogDec; MoCA < 26, *n* = 39). The median disease severity stage, according to the Hoehn and Yahr Scale (Hoehn and Yahr, 1967), was 2 for both groups, indicating that most patients were in the early to moderate stages of the disease. The mean disease duration was 8 years for the PD_CogInt group and 7.41 years for the PD_CogDec group [*t*_(66)_ = −0.53, *p* = 0.60]. All participants were undergoing anti-Parkinsonian pharmacological treatment (carbidopa/levodopa, D2 agonists, MAO inhibitors, amantadine, and/or anticholinergic agents) and exhibited a stable response to medication).

A total of 33 neurologically healthy controls (HC) were included for statistical comparison. They were recruited from the Juan XXIII Senior Center in Móstoles and the University for Older Adults Program (PUMA) at the Autonomous University of Madrid (UAM). Common exclusion criteria for both patients and HC included a diagnosis of major depression according to DSM-5 criteria [American Psychological Association (APA), 2014], a history of comorbid neurological conditions, and uncorrected significant visual or hearing impairments. Groups were matched for age and gender, and did not differ significantly on scores from the Spanish version of the Geriatric Depression Scale-Reduced form (GDS-R; Izal et al., 2010; see Table 1). As expected, ANOVA revealed significant differences in MoCA scores across groups [*F*_(2, 102)_ = 79.96, *p* < 0.001). Bonferroni *post hoc* tests showed that the PD_CogDec group (M = 22.10, SD = 3.00) scored significantly lower (*p* < 0.001) than both the PD_CogInt group (M = 27.84, SD = 1.49) and the HC group (M = 27.42, SD = 1.30).

**Table 1:** Descriptive variables and general cognitive/affective performance for PD_CogInt, PD_CogDec, and HC groups.

**Descriptive variables**	**PD_CogInt (M ±SD)**	**PD_CogDec (M ±SD)**	**HC (M ±SD)**	***X*^2^ or *F***	***p***
Age (Years)	64.90 ± 7.64	68.15 ± 9.76	63.97 ± 4.68	2.90	0.06
Gender (female), *n* (%)	13 (41.9)	9 (23.1)	14 (42.4)	5.21	0.27
MoCA	27.84 ± 1.49	22.10 ± 3.00	27.42 ± 1.30	79.96	< 0.001
GDS	1.30 ± 1.40	2.25 ± 2.07	1.42 ± 1.68	2.36	0.10

GDS, Geriatric Depression State; HC, Healthy controls; M, Mean; MoCA, Montreal Cognitive Assessment; PD_CogDec, PD patients with cognitive decline; PD_CogInt, Cognitively intact PD patients; SD, Standard Deviation.

Participants were informed of the confidential and anonymous treatment of their data and signed the informed consent. The study was completed in accordance with the Helsinki Declaration and approved by the Ethical Committee of the Universidad Autonoma de Madrid (Spain).

### 2.2 Instruments and procedure

Each participant completed standardized tests to assess his/her cognitive performance. The neuropsychological tests administered were categorized into five groups: processing speed, visuospatial abilities, memory, language and executive functions, and are recorded in detail in Table 2. The number of tasks used to assess each cognitive domain varied depending on the complexity and multidimensionality of the domain in question. For instance, executive functions were evaluated through several tasks targeting distinct but interrelated components such as cognitive flexibility, inhibition, and working memory. Likewise, for the memory domain, we included separate measures for verbal and visual memory to account for modality-specific processes. Our goal was to capture the full breadth of each domain's functioning, rather than to impose an artificial symmetry in the number of tests across domains. While this approach may result in an unequal distribution of tasks, we believe it provides a more comprehensive and ecologically valid assessment of cognitive performance.

**Table 2:** Cognitive assessment protocol.

**Cognitive process**	**Test**
Attention	Direct Digit WAIS-IV (DD; Wechsler, 2012)
Visuoperceptual ability	Benton Facial Recognition Test (BFRT; Benton et al., 1983)
Visuospatial ability	Judgment of Line Orientation Test (JLOT; Benton et al., 1978)
Memory	Spain-Complutense Verbal Learning Test (TAVEC; Benedet and Alexandre, 1998) 7_24 Recall Test (7_24RT; Barbizet and Cany, 1968)
Executive function	Stroop Color-Word Test (Stroop; Golden, 2001) Trail Making Test B-A (TMTB-A; Reynolds, 2002) Backward Digit WAIS-IV (BD; Wechsler, 2012) Phonemic Fluency tests (PFT; Benton and Hamsher, 1978)
Language	Boston Naming Test (BNT; Kaplan and Weintraub, 1983)

Empathy was evaluated using two instruments: one to measure dispositional (self-reported) empathy and the other to measure situational empathy. Self-reported empathy was evaluated by the Interpersonal Reactivity Index (IRI; Davis, 1983), one of the most commonly used measure of empathy. This scale is easy to apply and is composed of 28 items grouped into four subscales: Perspective Taking, Fantasy, Empathic Concern, and Personal Distress, each consisting of 7 items. It can measure both cognitive and emotional empathic reactions. The subscales Perspective Taking and Fantasy evaluate the more cognitive aspects and the subscales Empathic Concern and Personal Distress measure an individual's emotional reaction when faced with the negative experience of others.

The task designed to assess situational empathy consists of 20 videos depicting emotional situations (happiness, anger, fear, sadness, and neutral) that are potentially empathy-inducing (four from each of the emotions). Each video presents a dynamic emotional facial expression accompanied by a spoken sentence describing a daily-life situation related to the corresponding emotional expression. For example, a happy facial expression appears while the phrase “Congratulations, you're hired” is spoken. Participants were asked to describe how the situation made them feel by choosing from a set of five emotions the one that best described the emotion the situation evoked in them. Dynamic facial expressions were selected from the Crowd-sourced emotional multimodal actor's dataset (CREMA-D; Cao et al., 2014). Auditory stimuli were created and validated for this project by our research group from a database of statements with neutral and emotional contents created by Russ et al. (2008).

Participants were assessed independently in a quiet room. Both tasks were computerized and administered using a monitor at a visual distance of 60 cm. The E-Prime 2.0 software (Schneider et al., 2002) controlled the presentation of stimuli, the randomization of trials, and the recording of responses. The PD groups were evaluated at a time of day when their symptoms were less severe (“on-state”). The study was conducted in two separate sessions, each lasting 60 min, separated by ~1 week. The first session began with the collection of sociodemographic and clinical data, followed by the cognitive screening, and the first part of the tests to assess cognitive processes. In the second session, participants completed the second part of the cognitive processes tests and the two empathy tasks. To control for order effects, the presentation order of the two empathy tasks (situational and dispositional) was randomized across participants. Additionally, the 20 video clips used in the situational empathy task were presented in a randomized order for each participant.

### 2.3 Statistical analysis

In order to assess in depth cognitive background, one ANOVA was performed of each neuropsychological test. To assess dispositional empathy, one MANOVA 3 (Group: PD_CogInt, PD_CogDec and HC) x 4 (Dimension: Perspective Taking, Fantasy, Empathic Concern and Personal Distress) were carried out, with the number of correct responses as the dependent variable. To assess situational empathy, one ANOVA was carried out with correct responses as dependent variable for the three groups. Finally, Pearson correlations were used to assess the relationship between the two empathy measures and specific cognitive processes. Although some normality tests (e.g., Kolmogorov-Smirnov) yielded significant results, visual inspection of the histograms indicated that the distributions of the variables did not deviate substantially from normality. Given the relatively large sample size (*N* = 103) and the approximate symmetry of the distributions, parametric analyses were considered appropriate, following recommendations that support their robustness under mild deviations from normality (Norman, 2010).

## 3 Results

### 3.1 Cognitive background

Differences between the three groups in the cognitive tests were analyzed by performing a unifactorial ANOVA. As can be seen in Table 3, results showed differences between groups in all of them except for the Facial Benton, Stroop and PFT tests (*p* > 0.05). Bonferroni multiple comparisons tests revealed that PD_CogDec performed worse than HC for JOL, BNT, TAVEC, 7/24SR, DD, BD, and TMTB-A. For its part, PD_CogInt performed worse than HC for JOL, TAVEC, 7/24ST, and BD. Moreover, PD_CogDec also performed worse than PD_CogInt for JOL, BNT, BD, and TMTB-A.

**Table 3:** Mean and standard deviation in cognitive tests for PD_CogInt, PD_CogDec, and HC groups.

**Cognitive process**		**HC (M ±SD)**	**PD_CogDec (M ±SD)**	**PD_CogInt (M ±SD)**	***F***	***p***	**Statistically significant *post hoc* comparisons**
Attention	DD	9.85 ± 2.65	7.34 ± 1.93	9 ± 1.68	10.33	< 0.001	HC>PD_CogDec^***^; PDCogInt>PDCogDec^*^
Visuoperceptual abilities	BFRT	9.79 ± 1.73	9.26 ± 4.36	10 ± 4.87	0.267	0.77	
Visuospatial abilities	JLOT	25.76 ± 3.18	18.33 ± 6.74	22.26 ± 4.04	17.96	< 0.001	HC>PD_CogDec^***^; HC>PD_CogInt^*^; PDCogInt>PDCogDec^*^
Memory	TAVEC	48.85 ± 6.76	37 ± 11.28	41.30 ± 7.85	14.36	< 0.001	HC>PD_CogDec^***^; HC>PD_CogInt^**^
	7/24RT	29.24 ± 5.79	22.45 ± 7.11	22.96 ± 7.28	9.81	< 0.001	HC>PD_CogDec^***^; HC>PD_CogInt^**^
Executive functions	Stroop	0.01 ± 5.51	−2.51 ± 8.54	6.19 ± 35.88	1.32	0.27	
	TMT B-A	32.52 ± 16.80	103.72 ± 78.72	62.86 ± 62.57	12.12	< 0.001	HC>PD_CogDec^***^; PDCogInt>PDCogDec^*^
	BD	7.70 ± 2.08	4.62 ± 1.74	6.04 ± 2.27	21.14	< 0.001	HC>PD_CogDec^***^; HC>PD_CogInt^*^; PDCogInt>PDCogDec^*^
	PFT	17.88 ± 4.58	15.86 ± 5.84	18.78 ± 5.31	2.19	0.12	
Language	BNT	58.64 ± 1.95	52.69 ± 5.87	55.83 ± 3.23	17.02	< 0.001	HC>PD_CogDec^***^; HC>PD_CogInt^*^; PDCogInt>PDCogDec^*^

7/24RT, 7_24 Recall Test; BD, Backward Digit; BFRT, Benton Facial Recognition Test; BNT, Boston Naming Test; DD, Direct Digit; HC, Healthy controls; JLOT, Judgement Line Orientation Test; M, Mean; PD_CogDec, PD patients with cognitive decline; PD_CogInt, Cognitively intact PD patients; PFT, Phonemic Fluency Test; Stroop, Color and World Test Stroop; TAVEC, Spain-Complutense Verbal Learning-Codification; TMT B-A, Trail Making Test B-A; SD, Standard Deviation.

^*^p < 0.05; ^**^p < 0.01; ^***^p < 0.001.

### 3.2 Empathy

#### 3.2.1 Self-reported empathy

Table 4 presents the means and standard deviations for the three groups across each of the IRI subscales and the total score (Perspective Taking, Fantasy, Empathic Concern, and Personal Distress). The results revealed no significant main effect for Group, *F*_(2, 100)_ = 0.92, *p* = 0.40, $\eta_{p}^{2}$ = 0.02, nor for the Group × Subscale interaction, *F*_(6, 198)_ = 1.30, *p* = 0.26, $\eta_{p}^{2}$ = 0.04.

**Table 4:** Mean and standard deviation in dispositional and situational empathy tasks for PD_CogInt, PD_CogDec, and HC groups.

**Empathy task**	**Cognitive process**	**HC (M ±SD)**	**PD_CogDec (M ±SD)**	**PD_CogInt (M ±SD)**
Dispositional	Perspective taking	18.76 ± 3.46	20.54 ± 3.99	21.06 ± 4.40
	Fantasy	20.09 ± 2.77	19.91 ± 2.49	20.10 ± 4.08
	Empathic Concern	22.42 ± 2.86	22.77 ± 3.23	23.06 ± 3.47
	Personal Distress	19.91 ± 2.64	19.77 ± 3.36	19.52 ± 3.46
Situational		15.36 ± 1.92	13.23 ± 3.15	13.87 ± 3.63

HC, Healthy controls; M, Mean; PD_CogDec, PD patients with cognitive decline; PD_CogInt, Cognitively intact PD patients; SD, Standard Deviation.

#### 3.2.2 Situational empathy

Table 4 also presents the means and standard deviations for the PD groups and the control group across each of the emotions assessed (happiness, sadness, fear, anger, and neutral) in the situational empathy task. The ANOVA revealed a significant main effect of Group, *F*_(2, 100)_ = 4.71, *p* = 0.01, $\eta_{p}^{2}$ = 0.09. *Post hoc* comparisons indicated that the PD_CogDec group performed significantly worse than the HC group (*p* = 0.01), while no significant differences were found between HC and PD_CogInt (*p* = 0.12).

#### 3.2.3 Relationship between dispositional and situational empathy

Table 5 presents the Pearson correlations obtained for each group between the measures of situational empathy and dispositional empathy (distinguishing between total scores and subscales). The results showed the absence of any significant correlations among the different measures in all three groups.

**Table 5:** Correlations between dispositional and situational empathy for PD_CogInt, PD_CogDec, and HC groups.

**Dispositional empathy**	**Situational empathy**		
	**HC**	**PD_CogDec**	**PD_CogInt**
Perspective taking	−0.10	−0.01	−0.03
Fantasy	−0.31	−0.05	0.19
Empathic Concern	−0.06	−0.11	0.22
Personal Distress	−0.23	−0.01	0.08
Total	−0.34	−0.07	0.18

HC, Healthy controls; M, Mean; PD_CogDec, PD patients with cognitive decline; PD_CogInt, Cognitively intact PD patients.

### 3.3 Relationship between empathy and specific cognitive domain

Finally, correlations between dispositional and situational empathy abilities, on the one hand, and specific cognitive processes, on the other, were analyzed using Pearson correlation coefficients calculated separately for each of the three groups (see Table 6). The findings indicated that dispositional empathy did not demonstrate a significant correlation with any particular cognitive measure in any groups. Conversely, situational empathy was significantly associated with TAVEC scores in both the HC group (*r* = 0.39, *p* = 0.02) and the PD_CogDec group (*r* = 0.49, *p* = 0.007).

**Table 6:** Correlation between situational and dispositional empathy and specific cognitive measures for PD_CogInt, PD_CogDec, and HC groups.

**Cognitive measures**	**Empathy**					
	**Dispositional**			**Situational**		
	**HC**	**PD_CogDec**	**PD_CogInt**	**HC**	**PD_CogDec**	**PD_CogInt**
DD	−0.10	0.26	−0.07	0.05	0.09	−0.28
BFRT	0.06	−0.03	0.23	−0.08	0.35	−0.06
JLOT	−0.14	0.002	−0.01	0.18	0.14	−0.02
TAVEC	−0.02	−0.06	−0.03	0.39^*^	0.49^**^	0.13
7/24RT	−0.18	−0.04	0.06	0.02	0.14	0.08
Stroop	0.12	0.32	−0.05	0.02	0.31	−0.38
TMT B-A	0.02	0.08	−0.08	−0.01	0.08	0.12
BD	−0.02	0.12	0.11	−0.11	−0.06	−0.24
PFT	−0.12	0.21	−0.38	0.05	0.08	−0.10
BNT	0.10	−0.05	0.16	0.10	0.36	0.10

7/24RT, 7_24 Recall Test; BD, Backward Digit; BFRT, Benton Facial Recognition Test; BNT, Boston Naming Test; DD, Direct Digit; HC, Healthy controls; JLOT, Judgement Line Orientation Test; PD_CogDec, PD patients with cognitive decline; PD_CogInt, Cognitively intact PD patients; PFT, Phonemic Fluency Test; Stroop, Color and World Test Stroop; TAVEC, Spain-Complutense Verbal Learning-Codification; TMT B-A, Trail Making Test B-A.

^*^p < 0.05; ^**^p < 0.01.

## 4 Discussion

The present study aims to explore the empathic ability of patients diagnosed with PD, in comparison with a sample of HC. Specifically, it distinguishes between dispositional and situational empathy, and examines the potential relationship between these two measures. Furthermore, the study analyses the relationship between these two empathy measures and specific cognitive processes, both in patients with cognitive decline and cognitively intact. The results show that cognitively impaired PD patients exhibit poorer situational empathy compared to the other groups. In contrast, no group differences emerge in dispositional empathy. No significant correlation is found between dispositional and situational empathy, nor are there consistent correlations between empathy and specific cognitive processes. These results warrant further in-depth analysis to better understand their implications.

Regarding situational empathy, the fact that significantly lower performance is observed in PD patients with cognitive decline suggests that this type of empathy may be more dependent on cognitive resources. In this sense, situational empathy refers to the ability to respond empathetically to specific, real-time social and emotional stimuli (Auné et al., 2015; Cuff et al., 2014; Fernández-Pinto et al., 2008). As such, it is inherently context-dependent and requires the rapid integration of multiple cognitive and affective processes. To successfully engage in situational empathy, individuals must dynamically interpret and respond to the emotional states of others, often based on subtle social cues such as facial expressions, tone of voice, body language, and situational context. This requires the involvement of a broad set of cognitive abilities, including attention, verbal memory, and executive control, among others. In PD patients, especially in patients experiencing cognitive decline, these cognitive processes can be impaired (Aarsland et al., 2021, 2017; Fang et al., 2020). Therefore, deficits in situational empathy observed in cognitively impaired PD patients may reflect the underlying deterioration of these higher-order cognitive functions (Alonso-Recio et al., 2021; Schmidt et al., 2017).

With regard to dispositional empathy, the fact that no impairments were observed in PD patients—regardless of whether they exhibited cognitive decline—suggests that this type of empathy may not be affected either by the disease itself or by the patient's cognitive status. This type of empathy refers to a stable and generalized tendency to respond empathetically in daily life and is typically assessed through self-report questionnaires that rely on introspective ability and an overall judgment of one's own behavior. The fact that PD patients, both with and without cognitive impairment, do not show differences compared to controls on this measure suggest that PD patients might maintain a relatively preserved perception of their own emotional responses. The presence of preserved dispositional empathy in PD patients has been confirmed by previous studies (see Alonso-Recio et al., 2021; Schwartz and Pell, 2017). However, the existing literature does not offer a unanimous consensus regarding this finding. Some studies have reported both generalized impairments (affecting affective and cognitive empathy) and specific deficits differentially impacting cognitive vs. affective empathy (Dodich et al., 2021; Martinez et al., 2018; Multani et al., 2019; Narme et al., 2013; Pomponi et al., 2016; Schmidt et al., 2017).

It is important to highlight that significant differences among the studies reviewed may account for the discrepancies observed in their results. For instance, some studies do not include a HC group, and instead evaluate dispositional empathy impairment by examining the proportion of patients scoring low on the administered measures (Dodich et al., 2021; Pomponi et al., 2016). Additionally, other research indicates that empathic deficits in PD patients are primarily evident in those at more advanced disease stages. Since our sample predominantly comprised patients in intermediate stages, the possibility that declines in empathic ability correlate with disease severity cannot be ruled out (Schmidt et al., 2017). Finally, in some studies, empathy assessments were conducted using informant-report measures completed by family members or caregivers, rather than relying on patients' self-reports. These recent findings are especially relevant when interpreting our results. In this regard, an alternative explanation for our results may be that the lack of group differences is not due to a preserved self-perception of emotional responses in PD patients, but rather to biases inherent in the self-report instrument. In this sense, informant-based evaluations can uncover empathic deficits that patients themselves may be unaware of or unable to accurately appraise, particularly in the presence of cognitive decline, which can impair self-awareness and metacognitive abilities. Consequently, the observed discrepancies between self-reported and informant-reported empathy underscore a key limitation of relying exclusively on self-report instruments. Caregiver or family member assessments may provide a more sensitive and objective measure of empathic functioning, capturing subtle or otherwise unrecognized impairments that are not reflected in patients' own evaluations. This suggests that a comprehensive assessment of situational empathy in PD should incorporate multiple perspectives to obtain a more accurate and nuanced understanding of empathic capacities.

In any case, the absence of a correlation between dispositional and situational measures serves to reinforce the notion that these phenomena represent discrete constructs, a notion that is in accordance with theoretical models that postulate a dissociation between the empathic trait (more closely associated with personality factors and exhibiting stability over time) and the empathic state (more dependent on context and the availability of cognitive resources) (Aparicio-Flores et al., 2020; Cuff et al., 2014). These models are supported by empirical studies showing that the specific empathic emotion experienced in a situation is not necessarily strongly related to one's dispositional tendency to empathize (Batson et al., 1987; Fabi et al., 2019). In healthy individuals, only a modest relationship between the two measures has been observed (Fabi et al., 2019; Zickfeld et al., 2017). Moreover, some neuroimaging studies suggest that brain activity related to empathy correlates more strongly with situational rather than dispositional empathy scores (Bufalari and Ionta, 2013; Lamm et al., 2011; Saarela et al., 2007; Singer et al., 2004). Therefore, assessing both situational and dispositional empathy together could offer a more complete insight into empathic abilities.

Finally, when the correlations between empathy tasks (situational and dispositional) and specific cognitive measures are analyzed, it is observed that, while dispositional empathy does not correlate with any cognitive measure, situational empathy does. However, in the latter case, we did not find any consistent relationship with any specific cognitive processes. A correlation was observed only between situational empathy and the verbal episodic memory task, and this was limited to healthy controls and PD patients with cognitive impairment, but was not present in cognitively preserved PD patients. This correlation could suggest that empathic ability depends, at least in part, on the ability to remember previous emotional interactions, given that those who perform better on the empathy task also tend to show higher verbal episodic memory performance, and vice versa. However, it is important to note that although PD patients with cognitive impairment perform worse than HC on the verbal episodic memory task, cognitively intact PD patients —who show preserved situational empathy—also display lower memory performance compared to HC, and do not significantly differ from cognitively impaired patients in this domain. Therefore, this isolated correlation is not sufficient to confirm that the reduction in situational empathy is driven by verbal memory decline.

The overall findings of the present study can be interpreted within the framework of the neurodegenerative changes that characterize PD, particularly; those that involve brain networks of social cognition and empathy. Dispositional empathy, being more closely related to stable traits, may rely more heavily on relatively preserved cortical structures in the early stages of PD (Kunst et al., 2019). Such structures include the medial prefrontal cortex and temporoparietal cortex, which are key regions of the default mode network. This network is involved in self-reflection, theory of mind, and stable social information processing (Frith and Frith, 2006; Spreng et al., 2009). Conversely, situational empathy entails real-time processing of social and affective cues, and this may be contingent on dynamic neural systems that facilitate the integration of emotional, perceptual, and cognitive information. These include the limbic system (e.g., the amygdala and the anterior cingulate gyrus), the anterior insula, and the orbitofrontal cortex, regions that undergo alterations in function and structure from the early stages of PD (Banwinkler et al., 2022; Conti et al., 2024; Criaud et al., 2016; Gleichgerrcht et al., 2010; Poletti et al., 2012; Wen et al., 2025). The progressive impairment of these regions by Lewy body accumulation, loss of mesocorticolimbic dopamine, and cholinergic dysfunction may provide a theoretical basis for the reduced situational empathic capacity in patients with cognitive impairment (Chaudhuri and Schapira, 2009).

Moreover, the results obtained in the present study suggest a close relationship between situational empathy (which assesses a purely affective component) and general cognitive processes, leading us to suggest a complex interplay between affective and general cognitive networks that are required in the generation of adaptive empathic responses. This supports the view of empathy as a distributed phenomenon in the brain, requiring the integration of multiple higher-order cognitive processes (Zaki and Ochsner, 2012). Different neuroimaging studies point to the insula, a structure that appears affected in PD and that has been linked both to motor and non-motor symptoms, as a key structure in the integration of cognitive and emotional processes (Criaud et al., 2016; Gu et al., 2013).

From a clinical perspective, these findings highlight the importance of conceptualizing SC as a distinct and clinically relevant domain, whose systematic assessment may complement and enhance traditional neuropsychological evaluations in PD. This is particularly relevant given that impairments within this domain can significantly affect patients' relational functioning and emotional wellbeing (Christidi et al., 2018; Trompeta et al., 2021). More specifically, the results underscore the need to assess empathy in its multiple dimensions, particularly, in PD patients with suspected cognitive decline. In these cases, the identification of a specific deficit in situational empathy in patients with cognitive impairment may serve as an early clinical indicator of social functional impairment. Such deficits can contribute to challenges in social communication, increased caregiver burden, and diminished overall quality of life. Patients with reduced situational empathy may struggle to interpret emotional cues during dynamic interactions, leading to miscommunication, and social withdrawal. This, in turn, can intensify feelings of loneliness and isolation—already prevalent among individuals with PD (Prell et al., 2023; Terracciano et al., 2023).

Taken together, these findings suggest that situational empathy tasks may be especially valuable in identifying subtle social-cognitive impairments that are not detected by self-report measures. Although dispositional scales provide important insights into relatively stable socio-cognitive traits, they may be less sensitive to the dynamic, context-dependent challenges that patients face in everyday interactions. Therefore, we propose that situational empathy tasks may complement traditional empathy assessments by offering greater ecological validity and capturing clinically relevant deficits. This could be particularly useful when assessing patients with suspected cognitive decline, where early detection of functional impairment is crucial. Moreover, the absence of significant group differences in self-reported empathy suggests that exclusive reliance on subjective instruments may lead to an overestimation of patients' empathic capacities in everyday contexts. Additionally, these findings highlight the need to develop targeted affective intervention programmes, which may be especially beneficial for patients with mild to moderate impairment—prior to the onset of more widespread functional decline. In light of the observed associations between situational empathy and specific cognitive domains, combined cognitive-affective interventions—such as cognitive stimulation protocols enriched with social-cognitive components—could offer a more integrated and effective therapeutic approach.

Limitations of the study include the sample size, which, despite the homogeneity of the groups, may have restricted the statistical power to detect more subtle relationships. Additionally, the use of a single task for each type of empathy limits the generalizability of the results. Another important limitation of our study concerns the operationalization of empathy components. While our assessment of dispositional empathy included both affective and cognitive dimensions, our situational empathy task focused exclusively on the affective component. This asymmetry restricts the scope of our conclusions regarding the full empathic profile of the participants. Further research could fruitfully explore the intersection of these definitional approaches—for example, by investigating whether deficits in situational empathy are primarily driven by impairments in cognitive, affective, or both dimensions, and whether these patterns vary according to patients' clinical characteristics. Such investigations could help refine our understanding of empathy as a multidimensional construct and guide more targeted clinical assessments and interventions. On the other hand, although the situational empathy task aimed to simulate realistic, everyday scenarios, it still falls short of fully capturing the complexity of real-life empathic experiences. Therefore, future studies should aim to develop and incorporate more ecologically valid contexts capable of eliciting genuine empathic responses. Future studies may also benefit from a multimodal approach, including neuropsychological tasks, physiological measures, and neuroimaging, to better understand the mechanisms underlying situational empathy in PD. Finally, subsequent research efforts could explore how situational and dispositional empathy manifest across the various stages of Parkinson's disease.

## Data Availability

The datasets presented in this study can be found in online repositories. The names of the repository/repositories and accession number(s) can be found below: https://doi.org/10.5281/zenodo.15583909.
